# Temporal trends in disease-specific causes of cardiovascular mortality amongst patients with cancer in the USA between 1999 and 2019

**DOI:** 10.1093/ehjqcco/qcac016

**Published:** 2022-04-18

**Authors:** Zahra Raisi-Estabragh, Ofer Kobo, Phillip Freeman, Steffen E Petersen, Louis Kolman, Robert J H Miller, Ariel Roguin, Harriette G C Van Spall, Jacqueline Vuong, Eric H Yang, Mamas A Mamas

**Affiliations:** William Harvey Research Institute, NIHR Barts Biomedical Research Centre, Queen Mary University of London, London, UK; Barts Heart Centre, St Bartholomew's Hospital, Barts Health NHS Trust, West Smithfield, London EC1A 7BE, UK; Department of Cardiology, Hillel Yaffe Medical Center, Hadera, Israel; Keele Cardiovascular Research Group, Centre for Prognosis Research, Keele University, Keele, UK; Cardiology Department, Aalborg University Hospital, Hobrovej 18-22, 9100 Aalborg, Denmark; William Harvey Research Institute, NIHR Barts Biomedical Research Centre, Queen Mary University of London, London, UK; Barts Heart Centre, St Bartholomew's Hospital, Barts Health NHS Trust, West Smithfield, London EC1A 7BE, UK; Health Data Research UK, London, UK; Libin Cardiovascular Institute of Alberta, Calgary, AB, Canada; Libin Cardiovascular Institute of Alberta, Calgary, AB, Canada; Department of Cardiology, Hillel Yaffe Medical Center, Hadera, Israel; Department of Health Research Methods, Evidence, and Impact, Department of Medicine, Population Health Research Institute, Research Institute of St . Joe's, McMaster University, Hamilton, ON, Canada; UCLA Cardio-Oncology Program, Division of Cardiology, Department of Medicine, University of California, Los Angeles, CA, USA; UCLA Cardio-Oncology Program, Division of Cardiology, Department of Medicine, University of California, Los Angeles, CA, USA; Keele Cardiovascular Research Group, Centre for Prognosis Research, Keele University, Keele, UK; Department of Cardiology, Thomas Jefferson University, Philadelphia, PA, USA; Institute of Population Health, University of Manchester, Manchester, UK

**Keywords:** Cardio-oncology, Cancer, Cardiovascular disease, Cardiovascular mortality, Epidemiology, Mortality trends

## Abstract

**Aims:**

We report disease-specific cardiovascular causes of mortality among cancer patients in the USA between 1999 and 2019, considering temporal trends by age, sex, and cancer site.

**Methods and results:**

We used the Multiple Cause of Death database, accessed through the Centers for Disease Control and Prevention Wide-Ranging Online Data for Epidemiologic Research resource. We included 629 308 decedents with cardiovascular disease (CVD) recorded as the primary cause of death and active malignancy listed as a contributing cause of death. We created disease-specific CVD categories and grouped cancers by site. We calculated the proportion of CVD deaths attributed to each disease category stratified by sex, age, and cancer site. We also examined disease-specific temporal trends by cancer site. Ischaemic heart disease (IHD) was the most common cardiovascular cause of death across all cancer types (55.6%), being more common in men (59.8%), older ages, and in those with lung (67.8%) and prostate (58.3%) cancers. Cerebrovascular disease (12.9%) and hypertensive diseases (7.6%) were other common causes of death. The proportion of deaths due to heart failure was greatest in haematological (7.7%) and breast (6.3%) cancers. There was a decreasing temporal trend in the proportion of cardiovascular deaths attributed to IHD across all cancer types. The proportion of deaths due to hypertensive diseases showed the greatest percentage increase, with the largest change in breast cancer patients (+191.1%).

**Conclusion:**

We demonstrate differential cardiovascular mortality risk by cancer site and demographics, providing insight into the evolving healthcare needs of this growing high-cardiovascular risk population.

## Graphical Abstract

**Graphical Abstract:**
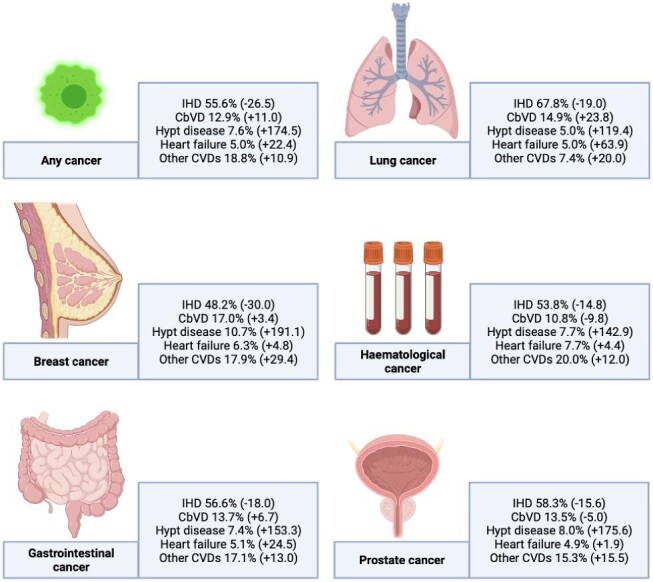
The proportion of cardiovascular deaths attributed to specific CVDs with temporal change from 1999 to 2019 shown in brackets. CbVD, cerebrovascular disease; CVD, cardiovascular disease; Hypt, hypertension; and IHD, ischaemic heart disease.

## Introduction

Improvements in cancer therapies have increased the life expectancy of patients with cancer.^1^ These individuals constitute a growing cohort at heightened cardiovascular risk,^2^ owing to the presence of shared risk factors,^3^ the pathophysiology of the underlying cancer,^4^ and a myriad of adverse cardiovascular effects of chemoradiation and other targeted therapies.^5^ Optimizing the cardiovascular health of cancer patients is an increasing healthcare priority requiring special considerations. In recent years, cardio-oncology has emerged as a new subspecialty^6^ and multiple international societies have published position statements dedicated to the cardiovascular care of cancer patients.^7–9^

There are significant differences in the types of cardiovascular diseases (CVDs) associated with different cancer sites,^2^ due in part to differences in demographics of patient populations and in the cancer treatments to which they are exposed. Furthermore, the burden from different CVDs is likely to change with the evolution of both cardiovascular and cancer treatments. For instance, the recent widespread use of novel cancer therapies with potential cardiovascular consequences, such as tyrosine kinase inhibitors in chronic myeloid leukaemia,^10^ trastuzumab in breast cancer,^11^ proteasome inhibitors in multiple myeloma,^12^ and vascular endothelial growth factor (VEGF) signalling pathway inhibitors for treatment of gastrointestinal and genitourinary cancers,^13^ is expected to impact CVD patterns in cancer patients.

Understanding differential CVD patterns and their temporal trends in different cancer types is important for appropriate risk stratification and for informing service planning and provision. However, to date, epidemiologic approaches to understanding the changing burden of cardio-oncology are limited and lacking in essential details. We present disease-specific cardiovascular mortality statistics of cancer patients in the US population over the last two decades extracted from national death registry data, stratified by sex, age, and cancer site. Importantly, we demonstrate temporal trends for disease-specific cardiovascular causes of mortality for different cancer sites.

## Methods

### Setting and study population

We used the Multiple Cause of Death database accessed through the CDC WONDER (Centers for Disease Control and Prevention, Wide-Ranging Online Data for Epidemiologic Research).^14^ This database comprises mortality and population counts from all US counties between 1999 and 2019. The data are based on death certificates for US residents. Each death certificate contains a single underlying cause of death and up to 20 contributing causes, along with demographic data. The underlying cause of death is extracted from conditions entered by the physician on the cause of death section of the death certificate. Causes of death are recorded in accordance with the International Classification of Disease, Tenth Revision (ICD-10) across the entire period of study. Deaths of non-residents (e.g. non-resident aliens and nationals living abroad) are not captured. The number of deaths, crude death rates, age-adjusted death rates, and 95% confidence intervals for death rates can be obtained by cause of death, place of residence, age, race, sex, and year.

### Analysis sample

We included all decedents with CVD (I00–I99) as the underlying cause of death and any malignant neoplasm (C00–C97) entered as a contributing cause of death. We categorized CVDs into ischaemic heart disease (IHD, I20–I25), heart failure (I50), hypertensive diseases (I10–I15), cerebrovascular disease (I60–I69), and ‘other’ CVDs (including CVDs falling outside of the defined categories, e.g. arrhythmias, valvular heart disease, pulmonary embolism, and pericardial diseases). We considered the following cancer sites: lung (C34), gastrointestinal (C15–C26), prostate (C61), breast (C50), and haematological (C81–C96). These five cancer site categories were selected to capture the most prevalent cancers, as per the latest national statistics from the USA.^15^

### Statistical analysis

Analysis was performed using IBM SPSS version 26. We calculated the percentage contribution of each CVD category to the overall cardiovascular mortality burden of patients with malignancy as a contributing cause of death. Results were stratified by cancer site, sex (male or female), and age (<45 years, 45–64 years, or >65 years). We analysed trends in cardiovascular mortality in cancer patients categorized by cancer type between 1999 and 2019. For comparison with general population trends, CVD mortality distribution and temporal trends are also presented for the whole sample available in CDC WONDER, including primary CVD deaths in individuals with and without record of cancer.

## Results

### Population characteristics

We identified 629 308 deaths attributed primarily to CVD with malignant neoplasm recorded as a contributing cause of death (*Table 1*). Of these, 269 201 (42.8%) deaths were in women, 4547 (0.7%) were in individuals aged under 45 years old, and 559 766 (88.9%) were in those aged over 65 years old. The five cancer sites covered 72.8% of all cancers, comprising 13.8% (*n* = 86 668) lung, 17.8% (*n* = 111 837) gastrointestinal, 16.6% (*n* = 104 766) prostate, 11.4% (*n* = 71 485) breast, and 13.2% (*n* = 82 940) haematological cancers. Except for breast cancer, all other cancers were more commonly observed in men; the sex discrepancy was greatest with lung (62.7% men) and prostate (all men) cancers. For all cancers, CVD death was significantly more common in older (over 65 years) individuals.

**Table 1:** Baseline characteristics

	Any cancer	Lung	GI	Prostate	Breast	Haematological
Total sample^a^	629 308	86 668 (13.8%)	111 837 (17.8%)	104 766 (16.6%)	71 485 (11.4%)	82 940 (13.2%)
Men	360 107 (57.2%)	54 318 (62.7%)	62 117 (55.5%)	104 766 (100%)	921 (1.3%)	47 334 (57.1%)
Women	269 201 (42.8%)	32 350 (37.3%)	49 720 (44.4%)	NA	70 564 (98.7%)	35 606 (42.9%)
Under 45 years old	4547 (0.7%)	294 (0.3%)	432 (0.4%)	17 (<0.1%)	369 (0.5%)	1040 (1.2%)
45–65 years old	64 984 (10.3%)	13 681 (15.8%)	11 563 (10.3%)	3893 (3.7%)	5669 (7.9%)	8759 (10.6%)
Over 65 years old	559 766 (88.9%)	72 693 (83.9%)	99 841 (89.3%)	100 852 (96.2%)	65 447 (91.5%)	73 140 (88.2%)

GI, gastrointestinal; NA, zero.

^a^Eleven deaths have data missing on age.

### Cardiovascular causes of mortality in cancer patients by age and sex

#### Any malignancy and the general population

**Figure 1:**
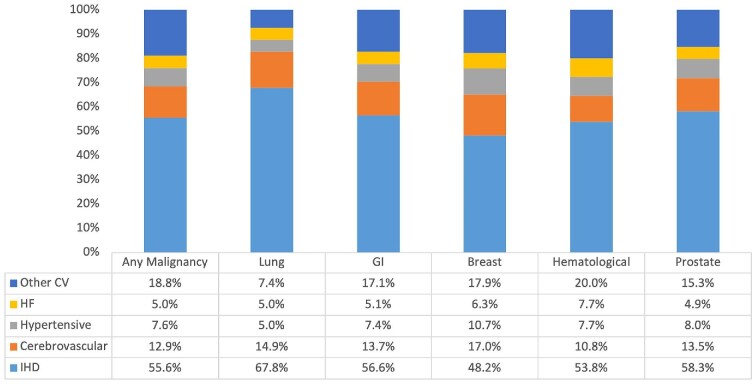
Disease-specific causes of cardiovascular mortality among cancer patients stratified by cancer site expressed as percentage of total cardiovascular mortality (1999–2019). CV, cardiovascular; GI, gastrointestinal; HF, heart failure; and IHD, ischaemic heart disease.

**Figure 2:**
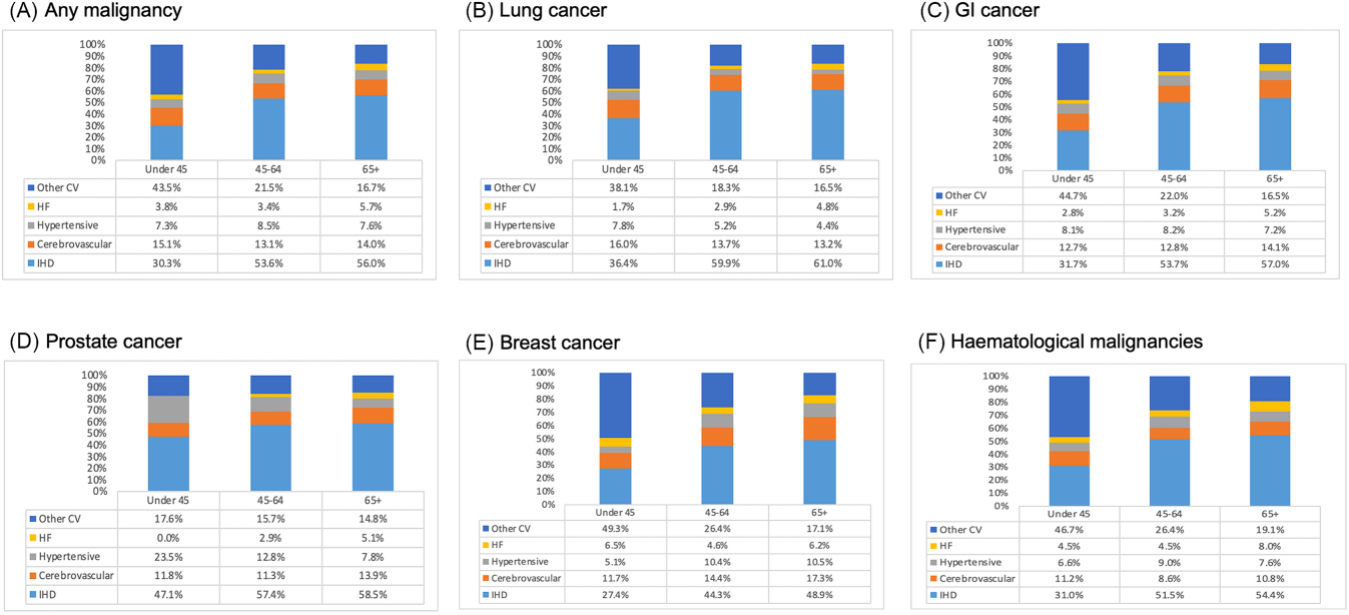
Disease-specific causes of cardiovascular mortality among cancer patients stratified by age and cancer site expressed as percentage of total cardiovascular mortality (1999–2019). GI, gastrointestinal; HF, heart failure; and IHD, ischaemic heart disease.

IHD accounted for the greatest proportion of cardiovascular deaths (55.6%) in patients who also had cancer listed as one of the contributing causes of death (*Table 2* and *Figure 1*). The remainder of deaths were split between cerebrovascular disease (12.9%), hypertensive diseases (7.6%), heart failure (5.0%), and other CVDs (18.8%). As expected, IHD accounted for a larger proportion of deaths in men (59.8%) than in women (49.8%). Accordingly, all other causes of death contributed larger proportions in women than men (*Table 2* and Supplementary material online, *Figure S1*). IHD (56.0% vs. 30.3%), hypertensive diseases (7.6% vs. 7.3%), and heart failure (5.7% vs. 3.8%) were more common causes of CVD death in individuals aged over 65 years old compared with those aged under 45 years (*Table 2* and *Figure 2A*). The proportion of deaths attributed to cerebrovascular disease appeared uniformly across all ages, being most common as a cause of death in those aged under 45 years old (*Table 2* and *Figure 2A*). The most common CVD causes of death in individuals under 45 years old were IHD (30.3%), cerebrovascular disease (15.0%), cardiomyopathies (10.0%), and pulmonary embolism and pulmonary heart disease (10.0%) (*Supplementary material online, Figure S2*).

**Table 2:** Cardiovascular causes of mortality among cancer patients presented as percentage of all cardiovascular mortality stratified by sex and age

				1999–2019			1999–2019, age (years)		
	1999	2019	Percentage change	Overall	Male	Female	<45	45–64	>65
**Whole sample^a^**									
Ischaemic heart disease	55.5	41.3	−25.7	48.9	54.1	43.9	33.2	53.5	48.5
Cerebrovascular disease	17.5	17.2	−2.2	16.9	14.0	19.8	14.1	12.7	17.8
Hypertensive diseases	4.5	11.7	+159	7.7	7.1	8.3	11.5	10.3	7.1
Heart failure	5.8	9.9	+71.2	7.5	6.5	8.5	2.4	3.3	8.5
Other cardiovascular disease	16.7	20.1	+20.1	19.0	18.4	19.5	38.7	20.2	18.1
**Any malignancy**									
Ischaemic heart disease	55.0	40.4	−26.5	55.6	59.8	49.8	30.3	53.6	56.0
Cerebrovascular disease	16.3	18.1	+11.0	12.9	12.2	16.3	15.1	13.1	14.0
Hypertensive diseases	5.5	15.1	+174.5	7.6	6.7	9.0	7.3	8.5	7.6
Heart failure	5.8	7.1	+22.4	5.0	5.0	6.1	3.8	3.4	5.7
Other cardiovascular disease	17.4	19.3	+10.9	18.8	16.3	18.9	43.5	21.5	16.7
**Lung cancer**									
Ischaemic heart disease	64.8	52.5	−19.0	67.8	64.5	54.0	36.4	59.9	61.0
Cerebrovascular disease	13.0	16.1	+23.8	14.9	11.0	17.0	16.0	13.7	13.2
Hypertensive diseases	3.1	6.8	+119.4	5.0	4.1	7.0	7.8	5.2	4.4
Heart failure	3.6	5.9	+63.9	5.0	4.1	5.0	1.7	2.9	4.8
Other cardiovascular disease	15.5	18.6	+20.0	7.4	16.3	17.0	38.1	18.3	16.5
**Gastrointestinal cancer**									
Ischaemic heart disease	61.0	50.0	−18.0	56.6	60.4	49.1	31.7	53.7	57.0
Cerebrovascular disease	13.5	14.4	+6.7	13.7	12.2	15.5	12.7	12.8	14.1
Hypertensive diseases	4.5	11.4	+153.3	7.4	6.6	7.5	8.1	8.2	7.2
Heart failure	4.9	6.1	+24.5	5.1	4.6	5.6	2.8	3.2	5.2
Other cardiovascular disease	16.1	18.2	+13.0	17.1	16.2	22.4	44.7	22.0	16.5
**Prostate cancer**									
Ischaemic heart disease	61.5	51.9	−15.6	58.3	58.3	—-	47.1	57.4	58.5
Cerebrovascular disease	13.9	13.2	−5.0	13.5	13.5	—-	11.8	11.3	13.9
Hypertensive diseases	4.5	12.4	+175.6	8.0	8.0	—	23.5	12.8	7.8
Heart failure	5.3	5.4	+1.9	4.9	4.9	—	0.0	2.9	5.1
Other cardiovascular disease	14.8	17.1	+15.5	15.3	15.3	—	17.6	15.7	14.8
**Breast cancer**									
Ischaemic heart disease	54.3	38.0	−30.0	48.2	—	48.2	27.4	44.3	48.9
Cerebrovascular disease	17.9	18.5	+3.4	17.0	—	17.0	11.7	14.4	17.3
Hypertensive diseases	5.6	16.3	+191.1	10.7	—	10.7	5.1	10.4	10.5
Heart failure	6.2	6.5	+4.8	6.3	—	6.3	6.5	4.6	6.2
Other cardiovascular disease	16.0	20.7	+29.4	17.9	—	17.9	49.3	26.4	17.1
**Haematological cancer**									
Ischaemic heart disease	56.8	48.4	−14.8	53.8	58.0	47.7	31.0	51.5	54.4
Cerebrovascular disease	12.3	11.1	−9.8	10.8	8.7	12.8	11.2	8.6	10.8
Hypertensive diseases	4.9	11.9	+142.9	7.7	6.7	9.2	6.6	9.0	7.6
Heart failure	6.8	7.1	+4.4	7.7	6.7	8.3	4.5	4.5	8.0
Other cardiovascular disease	19.1	21.4	+12.0	20.0	20.0	22.0	46.7	26.4	19.1

^a^Whole sample includes individuals with and without cancer code.

In the whole sample (general population), including individuals with and without record of cancer, IHD was also the most common cause of primary CVD death, although occurring less commonly (48.9% of all CVD deaths) than amongst individuals with record of malignancy (*Table 2*). As within the cancer cohort, we observed higher risk of IHD death amongst men than women (54.1% vs. 43.9%). In the general population, IHD death was most common among middle-aged individuals (45–64 years old), rather than among those over 65 years old (as in the cancer cohort). Cerebrovascular disease (16.9%), hypertensive diseases (7.7%), and heart failure (7.5%) were other common causes of primary cardiovascular death in the general population. As within individuals with record of malignancy, these deaths all occurred more commonly in women compared with men. Cerebrovascular disease and heart failure deaths occurred more commonly in older individuals (over 65 years) than in younger individuals.

#### Lung cancer

The contribution of IHD to cardiovascular deaths was greater among those with lung cancer (67.8%) entered as a contributing cause of death than in any other cancer type (*Table 2* and *Figure 1*). As consistent across all cancer types, the proportion of IHD deaths was higher in men (64.5%) than in women (54.0%). Cerebrovascular disease was the second most common cardiovascular cause of death among those with lung cancer, occurring more commonly in women (17.0%) than in men (11.0%). Similarly, hypertensive diseases were more common in women (7.0%) than in men (4.1%). Consistent with overall trends, IHD and heart failure deaths were progressively more common in older (over 65 years old) than in younger age categories (*Table 2* and *Figure 2B*). Cerebrovascular and hypertensive deaths were more common in the youngest (under 45 years old) age category (*Table 2* and *Figure 2B*).

#### Gastrointestinal cancer

Among those with gastrointestinal cancer recorded as a contributing cause of death, IHD (56.6%), cerebrovascular disease (13.7%), and ‘other’ CVDs (17.1%) were the top three causes of cardiovascular death (*Table 1* and *Figure 1*). IHD death was less common in women (49.1%) than in men (60.4%), while death due to all other diseases was more common in women. Deaths due to IHD, cerebrovascular disease, and heart failure all occurred more commonly in individuals aged 65 and above than in younger ages (*Table 2* and *Figure 2C*). In those aged <45 years old, IHD contributed 31.7% of deaths, while ‘other’ CVDs contributed 44.7% of deaths (*Table 2* and *Figure 2C**).*

#### Prostate cancer

Among those with prostate cancer entered as a contributing cause of death, 58.3% of cardiovascular deaths attributed to IHD, 13.5% to cerebrovascular disease, and 15.3% to other CVDs (*Table 2* and *Figure 1*). Deaths due to IHD, cerebrovascular disease, and heart failure were more common in individuals aged over 65 years old than in the younger age categories (*Table 2* and *Figure 2D*). Across all the subgroup analyses (cancer type, age, and sex) considered, the largest proportion of hypertensive disease deaths was among those aged under 45 years old with prostate cancer, comprising 23.5% of all cardiovascular deaths in this subset (*Table 2* and *Figure 2D*).

#### Breast cancer

Among individuals with breast cancer as a contributing cause of death, although IHD was the most common cause of cardiovascular death (48.2%), it occurred less frequently than in other cancer types (*Table 2* and *Figure 1*). Cerebrovascular disease (17.0%) and hypertensive diseases (10.7%) were more common causes of death in breast cancer than in any other cancer type (*Table 2* and *Figure 1*). IHD, cerebrovascular disease, and hypertensive disease deaths were more common in older (over 65) than younger ages (*Table 2* and *Figure 2E*). Among those with breast cancer, heart failure death (6.5%) was more common in those under 45 years old than in older age groups (*Table 2* and *Figure 2E*).

#### Haematological malignancies

Among those with haematological malignancies entered as a contributing cause of death, IHD was the most common cardiovascular cause of death and occurred more commonly in men (58.0%) than in women (47.7%). Death due to all other cardiovascular conditions occurred more commonly in women than in men (*Table 2* and *Figure 2F*). Heart failure deaths were more common among individuals with haematological malignancies than in all other cancer subtypes (7.7% vs. 5.0% overall). Women with haematological malignancies had the highest rates of heart failure death (8.3%), compared with any of the other sex, age, or cancer site stratifications examined (*Table 2*). IHD, hypertensive diseases, and heart failure deaths were more common in individuals aged over 65 years old than in younger ages. In those aged under 45 years old, cerebrovascular disease death (11.2%) and death from other CVDs (46.7%) occurred more commonly than in the older age categories.

#### Temporal trends in cardiovascular causes of mortality

**Figure 3:**
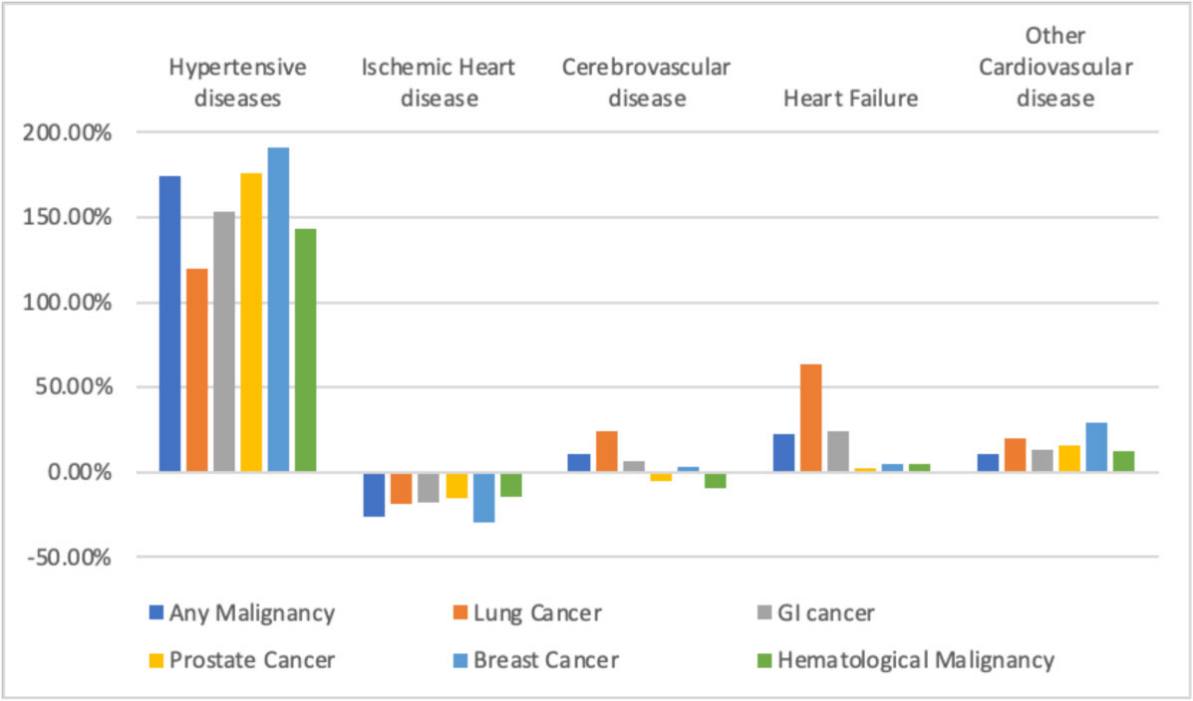
Percentage change in main causes of cardiovascular disease mortality in cancer patients, expressed as a percentage of total cardiovascular mortality by cancer site 1999–2019.

From 1999 to 2019, among individuals with any malignancy entered as a contributing cause of death, IHD accounted for a declining proportion of total cardiovascular deaths (from 55.0% to 40.4%), while the proportions of deaths from cerebrovascular disease (16.3–18.1%), hypertensive diseases (5.5–15.1%), heart failure (5.8–7.1%), and other CVDs (17.4–19.3%) increased (*Table 2*, *Figure 3*, and Supplementary material online, *Figure S3A*). These trends were broadly consistent for all cancer types, except for cerebrovascular disease deaths, which declined in haematological (12.3–11.1%) and prostate (13.9–13.2%) malignancies (*Table 2*, *Figure 3*, and Supplementary material online, *Figure S3*).

The largest percentage increase in contribution to cardiovascular deaths in cancer patients was from hypertensive diseases, with +174.5% increase between 1999 and 2019; this pattern was consistent across all cancer types (range: +119.4% to +191.1%), with the lowest increase in lung and greatest increase among breast cancer patients (*Table 2*, *Figure 3*, and Supplementary material online, *Figure S3*). The greatest proportion increase in heart failure deaths was in lung cancer patients (+63.9%) and the smallest increases were among prostate, haematological, and breast cancers. The proportion of deaths attributed to IHD declined across all cancer types.

Among the whole cohort (general population), including individuals with and without record of cancer, we observed similar declines in IHD mortality over the same time period (from 55.5% to 41.3%). As with individuals with record of malignancy, the largest change was an increase in cardiovascular deaths from hypertensive diseases (from 4.5% to 11.7%). Heart failure deaths also increased in the general population (from 5.8% to 9.9%), but by a greater magnitude than among the cancer cohort (+71.2% vs. +22.4%). Notably, in contrast to trends in the cancer cohort, we observed a decline in cerebrovascular deaths in the general population (−2.2% vs. +11.0%).

## Discussion

### Summary of findings

In this study of cardiovascular causes of death among cancer patients in the USA between 1999 and 2019, we identified differential patterns and trends by sex, age, and cancer site. The most common causes of primary CVD death, among patients with cancer entered as a contributing cause of death, were IHD, cerebrovascular disease, and hypertensive diseases. IHD accounted for the largest proportion of cardiovascular deaths across all cancer types but occurred most frequently in those with lung and prostate cancer. In line with the general population, deaths due to IHD occurred more commonly in men and older ages and there was a declining trend of IHD mortality over the two decades studied (1999–2019).^16^ Notably, while primary cerebrovascular disease deaths increased amongst individuals with record of cancer, we observed the reverse (decreasing) temporal trend in the general population over the same period. In younger cancer patients (aged under 45 years old), while IHD remained the most common cause of CVD death, other causes of death (e.g. cerebrovascular disease, cardiomyopathies, pulmonary embolism, and pulmonary heart disease) were seen more commonly than in older ages. Heart failure death was most common among those with prostate, haematological, and breast cancer, likely reflecting both the patient demographics and the therapies used in these cancers. In both the general population and among individuals with any malignancy recorded as a contributing cause of death, primary heart failure deaths were more common in older ages. In contrast, among those with breast cancer entered as a contributing cause of death, heart failure mortality was most common in younger patients (under 45 years old). Although there were temporal increases in the proportion of heart failure deaths across all cancer types, these increases were the most modest among prostate, breast, and haematological cancers and greatest among those with lung cancer. Hypertensive deaths had the largest percentage increase, with the greatest increase being among breast cancer patients.

### Comparison with existing work

The differential CVD patterns and temporal trends in different cancer types observed in this study may be explained by differences in the demographics and risk factor profiles of patient populations, and variation in influence of different cancer therapies on cardiovascular health. Recently, Stoltzfuz *et al.* demonstrated a declining rate of CVD specific mortality rates in patients with cancer.^17^ However, it is worth noting that the standardized mortality ratios (relative to patients without cancer) were higher in patients with a more contemporary cancer diagnosis. Patients with a history of cancer admitted with a cardiovascular diagnosis are at higher risk of in-hospital mortality compared to patients without cancer.^18^ Sturgeon *et al.* demonstrated that the contribution of cardiovascular deaths to overall mortality varies significantly across cancer sites.^19^ However, details regarding the components of heart disease-related deaths were not available in their study.

The dominance of IHD as a cause of cardiovascular death in the study population, its greatest occurrence in men and older individuals, and its declining contributions to total cardiovascular death over the last two decades are in keeping with epidemiologic patterns in the general population, as reported in our study and by others.^20^ The proportion of cardiovascular deaths attributed to IHD was greatest amongst those with lung cancer entered as a contributing cause of death, accounting for over two-thirds of all cardiovascular deaths in these patients. This may be explained by the preponderance of men, older individuals, and smokers in this cohort, which are all major risk factors for both lung cancer and IHD. These patient-related factors are likely augmented by the greater likelihood of exposure of patients with lung cancer to mediastinal radiotherapy, which can initiate and accelerate atherosclerosis.^7^ Indeed, radiotherapy has been reported to increase the risk of myocardial infarction by two to seven folds, with the risk proportional to the radiation dose.^7^ These treatment effects likely have a significant risk-modifying effect. Although breast cancer patients may have similar radiotherapy exposure to those with lung cancer, IHD features less prominently as a cause of death, perhaps reflecting the dominance of younger women in the cohort and weaker links to smoking than lung cancer. Patients with prostate cancer entered as contributing cause of death had the second highest rate of IHD deaths; these patients comprised an older, male-only cohort with expected higher IHD risk. Furthermore, many of the androgen-blocking agents (androgen deprivation therapy, androgen receptor blockers, and androgen metabolism inhibitors) used in the treatment of prostate cancer have been linked to augmented risk of IHD.

In stratified analysis by cancer type, we found heart failure deaths to be most common among those with breast and haematological cancers as a contributing cause of death. The treatments used in these cancers likely play a major role in driving heart failure deaths. Breast cancer is commonly treated with anthracyclines and trastuzumab, both agents with potential short- and long-term cardiotoxicity.^7^ Notably, contrary to disease patterns in the rest of the cohort, breast cancer patients in the youngest age category were more likely to suffer heart failure death than those who were older, perhaps reflecting more intensive therapy choices in younger breast cancer patients with higher treatment doses or greater use of combination therapies,^21–23^ which carry augmented risk of cardiotoxicity. Further contributing factors to the risk of heart failure death in breast cancer patients include intersection of a number of shared risk factors such as hypertension, diabetes, and obesity.^24^

Heart failure deaths were most common among individuals with haematological cancers. This may reflect the frequent use of anthracycline agents for the treatment of haematological malignancies, such as leukaemia and lymphomas.^25–27^ Furthermore, the use of anthracyclines in induction therapy prior to bone marrow transplantation and complications such as graft-versus-host disease may carry long term elevated risks of cardiotoxicity.^28–30^ Our findings mirror previous work showing that the hazard ratio for developing heart failure compared with the age- and sex-matched controls is highest for patients with hematologic cancers, compared to other primary cancer sites.^2^ While anthracycline-induced cardiotoxicity can occur during treatment, it most commonly manifests within a year post-treatment, characterized by continuous and progressive decline in left ventricular function.^31^ Heart failure in these patients may go undetected, presenting several years later, often in the context of other precipitating events.^7^ The potential cardiotoxicity of anthracycline agents and trastuzumab are well documented and there are now established recommendations for active surveillance and targeted treatments for patients undergoing these treatments.^32^ Furthermore, awareness of the enhanced cardiovascular risk from coadministration of anthracyclines and trastuzumab has led to, where possible, separate administration of these agents in breast cancer patients.^33,34^ Although the proportion of heart failure deaths increased across all cancer types, the smallest increases were seen in those with prostate, breast, and haematological cancers. The greatest increase in heart failure deaths was among those with lung cancer (+63.9%); this may reflect improved survival from acute coronary events and greater time at risk to develop heart failure in this cohort; it is also likely that the aetiology of heart failure in lung cancer patients is related to other comorbidities and less directly driven by previous cancer therapies.

The largest percentage increase in contribution to cardiovascular deaths in cancer patients was from hypertensive diseases, with the greatest increase among breast cancer patients (+191.1% increase from 1999 to 2019). This trend may reflect demographic trends towards an ageing population, where survival to ages where end-organ damage from hypertensive diseases can cause death is occurring more commonly. The rising rates of hypertensive heart disease as cause of premature CVD death in the general population^35^ may be a further contributor to the observed trends in our study. Another key potential driver of these trends is the rising rates of obesity, which is a shared risk factor for both breast cancer and hypertension and may act as a common determinant of both conditions. Additionally, hormone receptor treatments are a major component of breast cancer therapy and although their specific role in driving hypertensive deaths is uncertain, they are known to increase cardiovascular morbidity and mortality amongst cancer survivors.^36^ A further consideration is the change in definition of hypertension proposed by the American College of Cardiology/American Heart Association in 2017,^37^ which lowered the threshold for diagnosis. This change will likely result in artefactual increase in the incidence of hypertension across the population, which would eventually impact mortality statistics. However, for chronic diseases such as hypertension, there is usually a lag in the impact of change in disease definitions on mortality trends, particularly as many years of exposure to hypertension would usually be expected prior to death due to the condition. Thus, although definitional changes may have produced artefactual increases in the rates of hypertensive disease deaths reported, these are likely to be small and are unlikely to explain the overall temporal trend observed in this study. While hypertensive deaths overall constitute a small proportion of total cardiovascular deaths (7.6%), the increasing trend in related deaths highlights the need for dedicated attention to blood pressure control in cancer patients, particularly those receiving cancer therapies with destabilizing effects. Further dedicated studies to elucidate more definitively the driving factors for this trend are needed.

### Clinical implications

We demonstrate differential cardiovascular mortality risk by cancer type and demographics and provide insights into the evolving healthcare needs of this growing high-cardiovascular risk population. Our work highlights the importance of cardiovascular risk factor modification in patients with cancer, who may benefit from targeted preventative therapies. Given the rising number of cancer survivors and the huge prognostic implications of cardiovascular disease in this cohort, the cardiovascular care of cancer patients is transitioning from an area with a dedicated specialist focus to having growing relevance to the general cardiologist. Indeed, cardiologists across all subspecialties will increasingly encounter cancer survivors with cardiovascular disease and understanding the patterns and risk profiles of such patients is highly important for appropriate treatment decisions. Further work is required to identify specific treatment gaps in optimization of major cardiovascular risk factors, such as hypertension, in this cohort. Additionally, exploration of cancer-specific cardiovascular risk profiles as well as programmes for mitigation of treatment-related cardiotoxicity is warranted. Although cancer and its related therapies are determinants of increased cardiovascular risk, CVD may also precede cancer diagnosis. As populations worldwide are getting older, such more complex clinical scenarios will be encountered with increasing frequency. Our work highlights the evolving healthcare needs of this increasing high-risk cohort from a population perspective and provides key insights into priorities for service planning and provision, which will likely extend beyond dedicated cardio-oncology services to wider aspects of general cardiology. There is need for continual review of the needs of patient populations and for proactive adaption of multi-disciplinary clinical services.

### Strengths and limitations

The use of national mortality data allowed for maximized coverage of an entire population with minimal sampling error. However, the data set excluded non-residents, and as such may not capture marginalized groups with poorer health outcomes compared with the general population. Recording of conditions using ICD-10 criteria allowed standardized ascertainment of both cancer and CVD. However, these codes may be subject to error from misdiagnoses or miscoding; such errors are likely to be occurring at random and are not expected to lead to systematic bias in the present analysis. It is possible that there is bias towards omission of cancer as a contributor to death in individuals with historic cancers, leading to underestimation of the long-term cardiovascular risk of cancer patients. Additionally, the overrepresentation of broadly less healthy individuals with more recent cancers or with greater clinical sequalae following cancer may have distorted patterns of cardiovascular mortality observed in our analysis. An important point to highlight is that the analysis sample in this study does not capture the total number of individuals with the various cancers studied, but rather the number of individuals who had record of ‘cancer as contributing cause of death’. Confirmation of the CVD mortality distributions and trends observed in our study using cohorts where the total number of cancer patients is more completely defined is warranted. Furthermore, as the aim of this work was to study cancer patients with CVD as the primary cause of death, our analysis does not include circumstances where CVD was a contributory cause of death in cancer patients. These are important considerations for capturing the full burden of CVDs amongst cancer patients and their incorporation into future research studies is warranted. Another limitation of our study is that we cannot be certain of the status of an individual's cancer. That is, we do not know the date of first cancer occurrence, recurrent disease, or whether the individual is undergoing active cancer treatment. Certain chemotherapeutic agents, targeted therapy, and/or radiotherapy are key determinants of cardiovascular risk in cancer patients. It was not possible to consider their direct modifying effects in the present data set. However, even if this information was available, isolating individual treatment effects in the presence of multiagent regimens and effects across multiple pathways is very challenging, which is further complicated given the heterogeneity of cancer based on genetic subtypes, stage of diagnosis, and treatment strategies, which may dynamically change. Finally, consideration of mortality outcomes provided robust endpoints for outlining the health burden of CVD in cancer patients. The current data set does not permit evaluation of the cardiovascular morbidity burden in this cohort; indeed, the pattern and incidence of non-fatal CVD may be different from the mortality trends described here. As life expectancy of cancer patients increases, the quality of life and time spent in good health are highly important and future studies addressing these questions are needed. Although we attempt to discuss our findings in the context of the existing literature, the great heterogeneity in study designs and the cohorts studied makes direct comparisons difficult. As this is a descriptive epidemiological study, our analysis is focused on describing the distribution and temporal trends in CVD mortality. We do not explicitly evaluate potential explanations for the observed trends. Thus, the interpretations suggested in the discussion of the results, at times, reflect potential explanations based on existing knowledge, rather than explicitly tested hypothesis. Furthermore, it is also possible that the observed disease trends in part reflect changes in disease definitions, clinical practice, and documentation of deaths over time. Analytic studies dedicated to examination of the potential drivers of the observed trends are needed.

## Conclusions

Our findings demonstrate differential patterns of disease-specific cardiovascular deaths across different cancer sites. Although IHD is the most common cause of cardiovascular death across all cancers, there is a declining trend in the proportion of deaths to which IHD is attributed. Indeed, in women and younger individuals, IHD is less dominant as a cause of cardiovascular death. Heart failure deaths were most common among patients with haematological and breast cancers, likely reflecting both the treatment factors and demographics of these patient populations. However, while heart failure deaths increased across all cancer types over the two decades studied, the smallest increases were in breast and haematological cancer patients, perhaps indicating both greater heart failure deaths in other cancers due to an ageing population and the success of cardiovascular programmes targeted at anthracycline and trastuzumab cardiotoxicity in breast and haematological cancer patients. Finally, hypertensive diseases are an increasing cause of cardiovascular deaths, highlighting the need for attention to blood pressure control of cancer patients. Thus, our findings demonstrate differential cardiovascular mortality by cancer site and provide insights into the evolving healthcare needs of the growing cancer and high-cardiovascular risk population.

## Supplementary Material

qcac016_Supplemental_File

## Data availability

The data underlying this article are available in the CDC WONDER database, at https://wonder.cdc.gov/.
