# Supplementary material for: Temporal trends in disease-specific causes of cardiovascular mortality amongst patients with cancer in the USA between 1999 and 2019
Source: Eur Heart J Qual Care Clin Outcomes. 2022 Apr 18;9(1):54–63. doi: 10.1093/ehjqcco/qcac016 (PMC9745666; doi:10.1093/ehjqcco/qcac016)
Supplement: qcac016_Supplemental_File [file qcac016_supplemental_file.docx]

**SUPPLEMENTARY MATERIALS**

**Supplementary Figure 1. Disease specific causes of cardiovascular mortality amongst male (A) and female (B) cancer patients stratified by cancer site expressed as percentage of total cardiovascular mortality (1999-2019)**

**
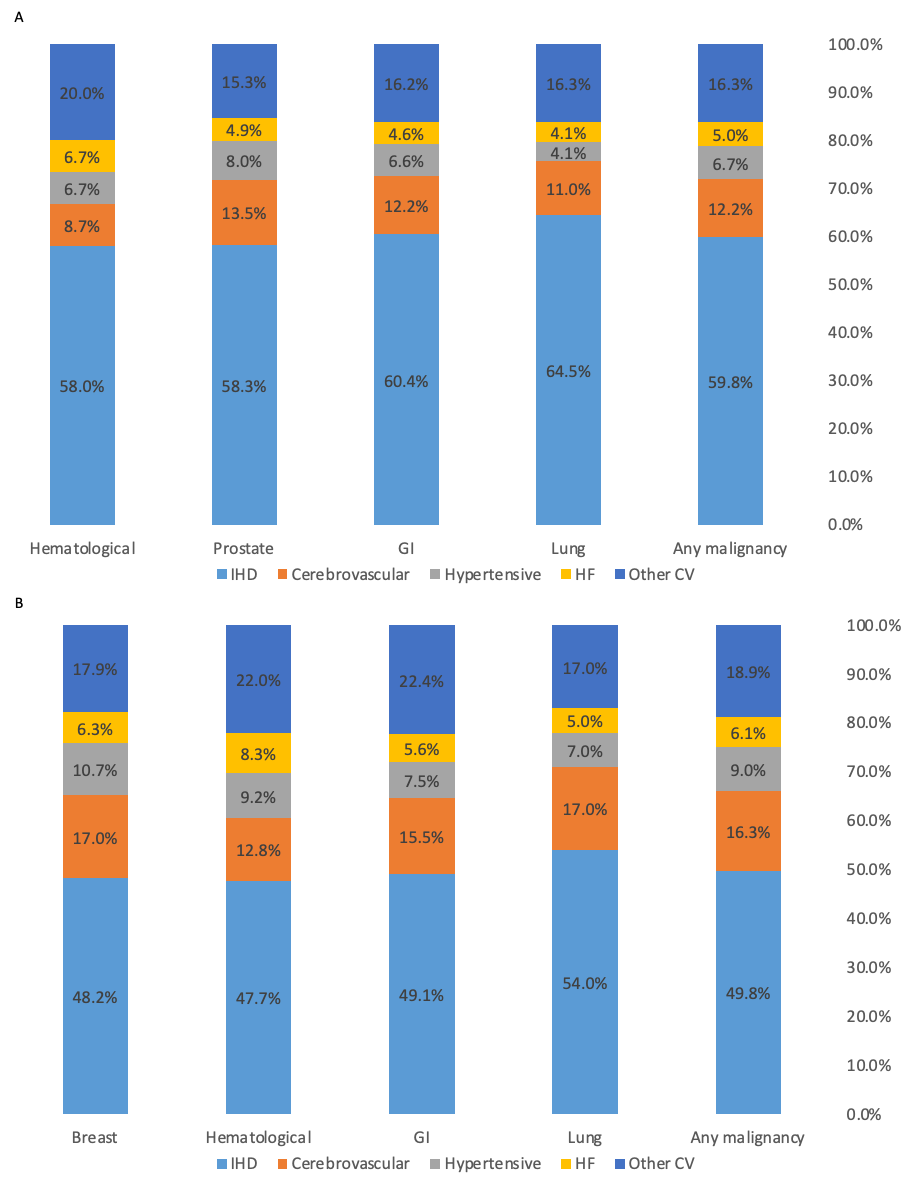
**

**Supplementary Figure 1 footnote.** CV: cardiovascular; GI: gastrointestinal; HF: heart failure; IHD: ischaemic heart disease

**Supplementary Figure 2. Distribution of CVD causes of mortality amongst those aged under 45 years old with record of any malignancy**


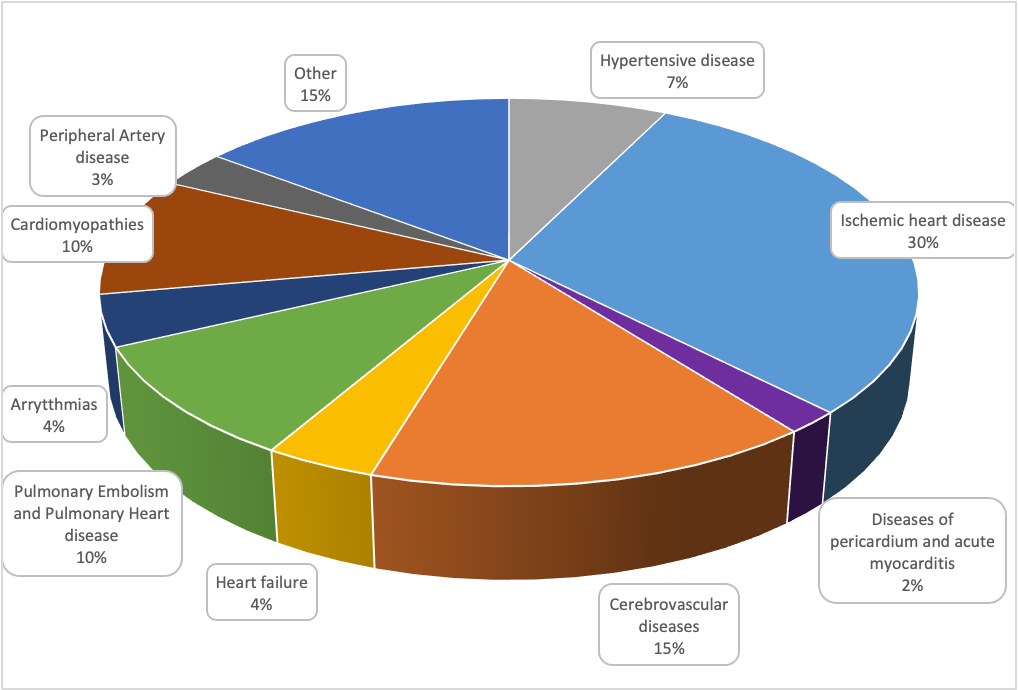


**Supplementary Figure 2 footnote.** CVD: cardiovascular disease.

**Supplementary Figure 3. Trends in disease specific causes of cardiovascular mortality amongst cancer patients stratified by cancer site expressed as percentage of all cardiovascular mortality**

A. Any malignancy

B. Lung cancer

C. GI cancer

D. Prostate cancer

E. Breast cancer

F. Haematological malignancies


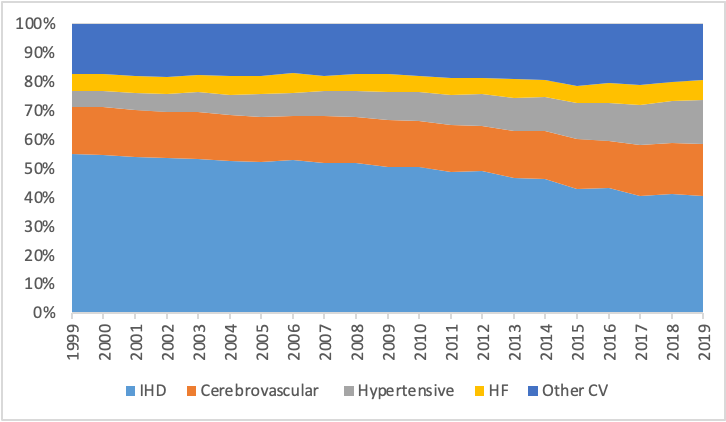

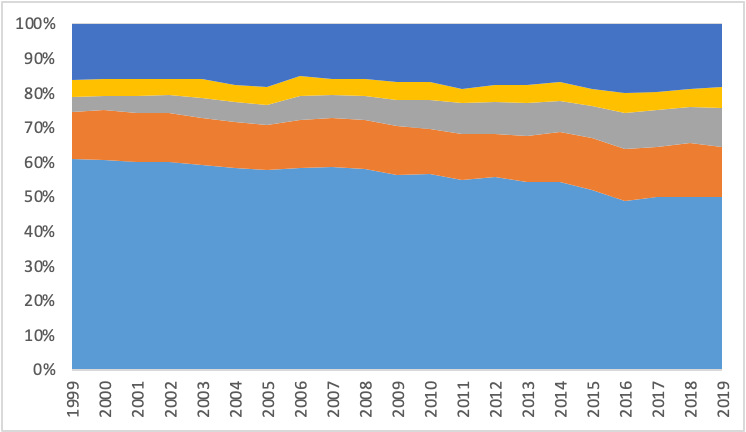

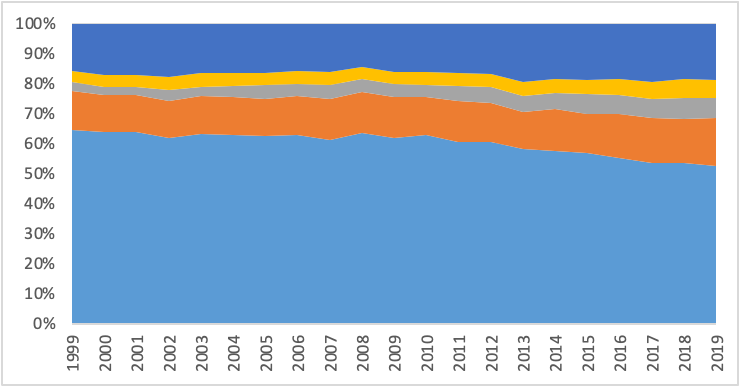

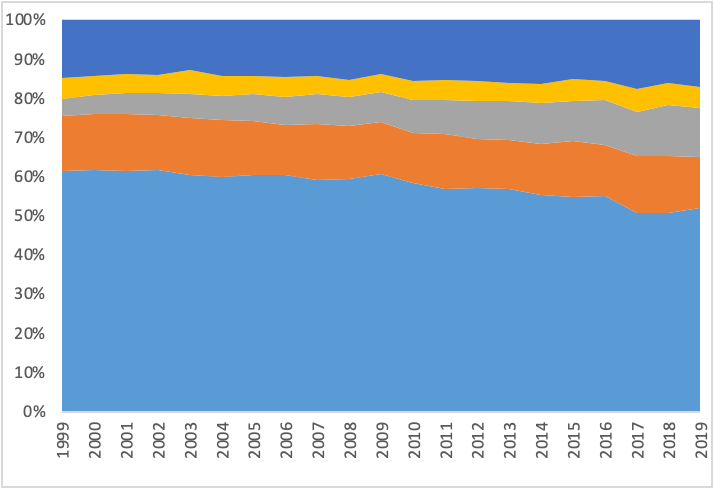

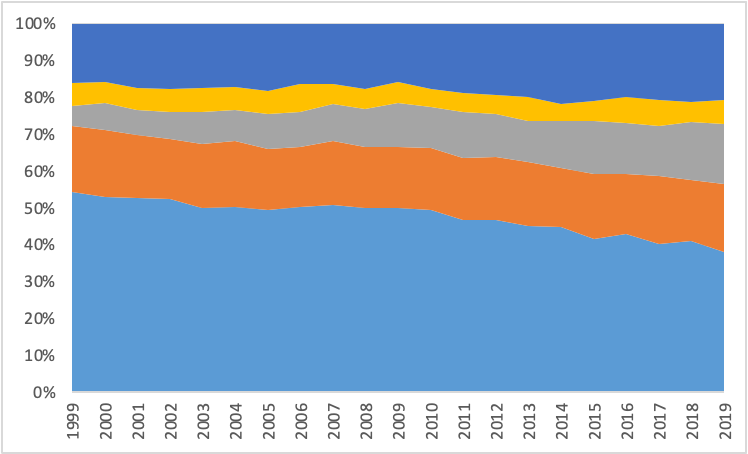

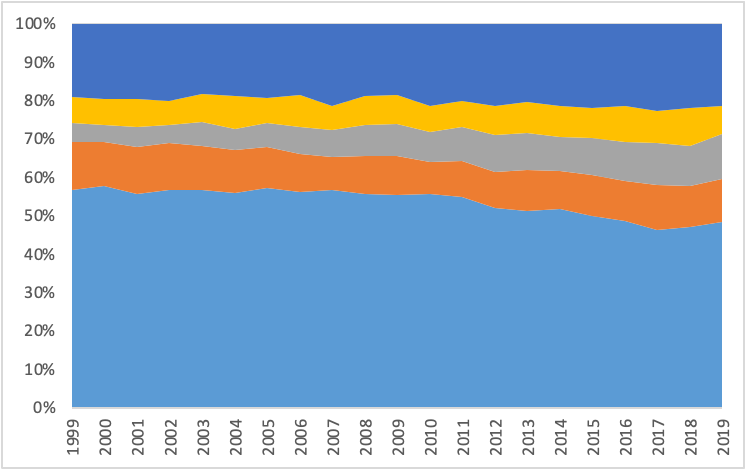


**Supplementary Figure 3 footnote.** GI: gastrointestinal; HF: heart failure; IHD: ischaemic heart disease.
